# The Thirty-third Commencement of the Buffalo Medical College

**Published:** 1880-03

**Authors:** 


### THE THIRTY-THIRD COMMENCEMENT OF THE BUFFALO MEDICAL COLLEGE.

The Medical Commencement was held Wednesday evening, February 25, at St. James Hall, and was attended by a large and appreciative audience. Prof. Thomas F. Rochester gave the address to the graduating class. His subject, "State Medicine," was exhaustively treated, and its discussion added much to the scholarly reputation, already acquired by the speaker, both at home and abroad. We hope to give abstracts from this valuable paper in a future number of the Journal. President Anderson, of the Rochester University, addressed the Alumni.

We give a complete list of the graduating class:

Clayton Moses Daniels, Buffalo, N. Y., Management of Natural Labor; Henry Warren Rogers, East Aurora, N. Y., Median

Lithotomy; George W. Reynolds, Lockport, N. Y., Infectious Diseases; Charles Anthony McBeth, Tonica, Ill., Ovulation; William Henry Allen, Waverly, N. Y., Scarlatina; Adelbert Theodore Bacon, Canaseraga, N. Y., Acute Gastritis; William Robertson Campbell, Trumansburg, N. Y., Dropsy; Eddy Smith Freeman, Middleport, N. Y., Spermatorrhœa; Teneyck Olmstead Burleson, Howard, N. Y., Amenorrhœa; Marcus Abner Dumond, Danby, N. Y., Typhoid Fever; Joseph Robert Love, Buffalo, N. Y., Philosophy of Practical Medicine vs. Empiricism; Myron Smith Watkins, Spencer, N. Y., Diphtheria; John Park Mason, Elmira, N. Y., Puerperal Eclampsia; Ransom Elbert Moss, Gowanda, N. Y., Acute Pneumonia; Frank Brockway, Cambria, N. Y., Dysmenorrhœa; Kay A. Sweet, Bradford, Pa., Gonorrhœa; AlfredBeers Kibbe, Buffalo, N. Y., Color Blindness; William Richards Laird, Trumansville, N. Y., Accoucheur; Edward Clark, West Seneca, N. Y., Fractures of Shaft of Femur; Adoniram Jay Kniffen, East Saginaw, Mich., Gonorrhœa; John Anthony Hoffmeyer, Buffalo, N. Y., Treatment of Phthisis Pulmonalis; Charles Menzies Briggs, West Macedon, N. Y., Physiology of Rest in Health and Disease; Samuel Henry Warren, Buffalo, N. Y., Sciatica; Alfred Milton Mead, Macedon Centre, N.Y., Urinary Examinations; Pardon Leland Kimball, Jamestown, N. Y., Scarlatina; Morris Pierce Pomeroy, Grand Rapids, Wis., Typhoid Fever; Vernon Mark Griswold, Cassadaga, N. Y., Inflammation; Carl Bronson Smith, Painted Post, N. Y., Operative Surgery; Cassius Oresta Jackson, Canandaigua, N. Y., Digestion; Fremont C. Knight, Arcade, N. Y., Persons Found Dead; Benjamin Daniel Collins, Suspension Bridge, N. Y., Pleuritis; Charles Gordon Champlin, Williamsville, N. Y., Pulmonary Tuberculosis; Clarence Eugene Griggs, Strykersville, N. Y., Scarlatina; Mary Jane Slaight, Scottsburgh, N. Y., Morbus Coxarius; Otto Appley, Damascus, Pa., Rubeola; Charles Dallas Shumway, Watertown, N. Y., Amenorrhœa; Carl Henry Guess, Buffalo, N. Y., Heart's Action; Frank Owen Vaughn, Buffalo, N. Y., Venereal Diseases; Fred Emerson Tuttle, Cassada, N. Y., Remittent Fever;

William Samuel Town, Cambria, N. Y., Talipes; Frank Manuel Gipple, Bowmansville, N. Y., Biliary Calculi; Joseph Morris Lewis, Elba, N. Y., Animal and Vegetable Physiology; Timothy Lowthian, Caro, Mich., Fractures; James Israel Northrop, Aylmer, Ont., The Blood; James Augustus Paulding, Drummondsville, Ont., Apoplexy; Eugene Clinton Waldurff, Clyde, N. Y., Disease; Henry Gould Chamberlain, Albion, N. Y., Spinal Irritation; Allen Stewart Smith, Cape Vincent, N. Y., Phlegmasia Dolens; William Cary Barrett, Buffalo, N. Y., Leucocythemia; Benjamin Hershey Grove, Buffalo, N. Y., Hemiplegia; Frank Kerst, Mecklenburg, N. Y., Acute Peritonitis; Mary Berkes, Eggertsville, N. Y., Taking Care of the Sick; Frederick Peterson, Buffalo, N. Y.

